# Safety of vaginal delivery in women with placental chorioangiomas diagnosed by prenatal ultrasound: A retrospective cohort study

**DOI:** 10.1097/MD.0000000000029672

**Published:** 2022-07-22

**Authors:** Jiashan Zou, Weimin Ding, Qin Chen, Xiaoxia Bai, Baohua Li

**Affiliations:** a Department of Obstetrics, Women’s Hospital, Zhejiang University School of Medicine, Xueshi Rd No. 1, Hangzhou 310006, Zhejiang, People’s Republic of China; b Zhejiang University School of Medicine, Hangzhou 310006, People’s Republic of China; c Department of Pathology, Women’s Hospital, Zhejiang University School of Medicine, Hangzhou 310006, People’s Republic of China.

**Keywords:** cesarean section, placental chorioangioma, pregnant outcomes, safety, vaginal delivery

## Abstract

This study aimed to examine the maternal and neonatal outcomes in different mode of delivery in pregnant women with placental chorioangiomas, in order to determine the safety of vaginal delivery.

We conducted a retrospective study of 54 women with placental chorioangioma diagnosed by prenatal ultrasound and subsequently proven histologically, excluding those who underwent cesarean section for obstetric indications. The mode of delivery was divided into a vaginal delivery group (23 women) and a cesarean section group (31 women). The indication of cesarean section group was only for placental chorioangioma, no other obstetric indications. The maternal characteristics, pregnancy outcomes and the color doppler imaging characteristics of placental chorioangioma of the 2 groups were compared, and the clinical characteristics of women in the vaginal delivery group were described in detail.

The incidence of placental chorioangioma was nearly 0.43‰ in our study. There was no significant difference in the maternal characteristics and pregnancy outcomes between the 2 groups. 82.6% (19/23) of the women successfully delivered vaginally and 4 failed who turned to cesarean section in the vaginal delivery group; among them, 17 women had giant chorioangiomas (>4 cm in diameter). The direct cause of vaginal delivery failure was fetal distress, persistent occiput posterior fetal position and cephalopelvic disproportion.

Pregnant women with placental chorioangiomas and no other obstetric indications for cesarean section may attempt a vaginal delivery, even with giant chorioangiomas. If there are risk factors of vaginal delivery failure, the progress of labor needs to be closely monitored.

## 1. Introduction

Chorioangioma is the most common nontrophoblastic vascular tumor of the placenta, with the incidence of 1%^[1,2]^ and was first described by Clarke in 1798.^[3]^ It is derived from primitive chorionic mesenchymal or placental hemangiomas arising from the placental blood vessels.^[4]^ Some researchers believed that the incidence of placental chorioangioma is related to altitude, and may be related to overexpression of vascular endothelial growth factor caused by hypoxia.^[5]^ The total incidence is underestimated as most placental chorioangiomas are single, small, symptomless, and difficult to be detected by prenatal ultrasound, and can only be found after careful, routine histologic examination of the placenta.^[6]^

Complications are mostly associated with chorioangiomas larger than 4 cm in diameter known as giant chorioangiomas,^[7]^ which are detected prenatally. Giant chorioangiomas are rare with an incidence ranging between 1/3500 and 1/9000 pregnancies.^[4,6]^ They may be associated with a series of pregnancy and fetal complications, leading to poor pregnancy outcomes, such as polyhydramnios, preeclampsia, premature rupture of the membranes, placental abruption, preterm labor, fetal growth restriction (FGR), fetal nonimmune hydrops, fetal anemia, and fetal death.^[4,8]^

In the event of associated complications during pregnancy, the mode of delivery should be considered in terms of fetal maturity and the available neonatal support. But the delivery mode of pregnant women with placental chorioangioma without obstetric indications for cesarean section has not been currently studied. In our previous study, we described the characteristics of women with placental chorioangioma who delivered in our hospital,^[9]^ but we did not assess the delivery mode and the safety of vaginal delivery for those patients. The objective of the study was to investigate the pregnancy outcomes in pregnant women with placental chorioangiomas without obstetric indications for cesarean section by different modes of delivery, in order to determine the safety of vaginal delivery. This study has potential clinical usefulness to assess the clinical characteristics, pregnancy outcomes, and the mode of delivery in the women.

## 2. Methods

**Figure 1.**
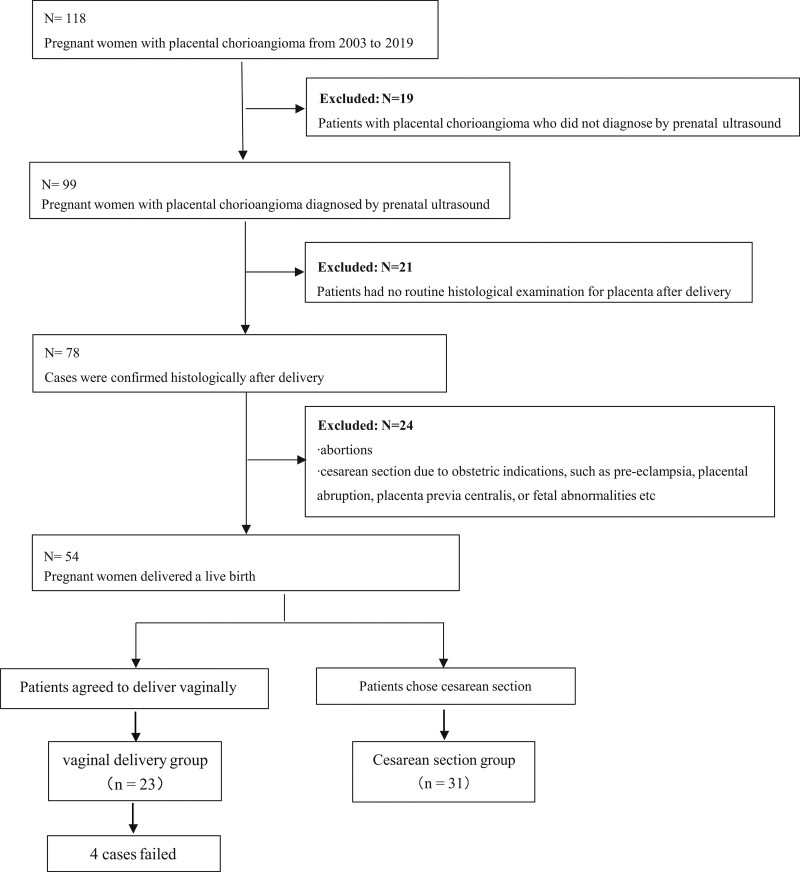
Flow diagram of the study.

We designed a retrospective cohort study using the clinical data of pregnant women with placental chorioangioma diagnosed by prenatal ultrasound, attending The Women’s Hospital, Zhejiang University School of Medicine in Hangzhou, China, between January 2003 and December 2019. The study was approved by the Institutional Review Board of Zhejiang University School of Medicine. Women were only eligible if they had delivered a live birth, and diagnosed by prenatal ultrasound and subsequently proven histologically, and women were excluded if they underwent abortions or cesarean section due to obstetric indications, such as preeclampsia, placental abruption, placenta previa centralis, or fetal abnormalities etc, or had no routine histological examination for placenta after delivery (Fig. 1).

Women who met the inclusion criteria were assigned to either a vaginal delivery or a cesarean section group, as follows: The vaginal delivery group consisted of patients who had agreed to deliver vaginally. This group included 4 women who failed to deliver vaginally and turned to cesarean section due to fetal distress, persistent occiput posterior fetal position and cephalopelvic disproportion. The cesarean section group consisted of women who underwent cesarean section only due to placental chorioangioma.

The prenatal diagnosis of placental chorioangioma was established by routine B-mode ultrasound examinations and color Doppler flow imaging. On sonography, the lesions showed a well-circumscribed placental mass with similar or higher echogenicity to the surrounding tissue and a clearly delineated vascular supply.^[10]^ Large tumors can be of variable shapes and divided by fibrous septa.^[11,12]^ All cases were confirmed histologically after delivery by a senior pathologist (Qin Chen).

Maternal characteristics and pregnant outcomes were collected for both the vaginal and cesarean section delivery groups by reviewing each patient medical records. The data included maternal characteristics (age, body mass index [BMI], gravidity, parity, method of fertilization), color doppler imaging (CDI) characteristics of placental chorioangioma (gestational age at diagnosis, location, blood flow, and size at the time of delivery) and pregnancy outcomes (gestational age at delivery, duration of labor, maternal and infant complications, postpartum hemorrhage, puerperal infection, neonatal birth weight, gender, and Apgar scores at 1 and 5 minutes).

All statistical analyses were performed with the Statistical Package for the Social Sciences Version 22.0 (IBM Corp., Armonk, NY). Descriptive analysis were performed by calculating the frequencies and percentages for categorical variables; means (and standard deviations) for continuous variables, if normally distributed; or the medians (ranges), if not normally distributed. The Chi-square test, Fisher exact test, *t*-test and the rank sum test were used, as appropriate, to compare data between the groups. *P* values of < 0.05 were considered to be statistically significant.

## 3. Results

### 3.1. Clinical characteristics

Between January 2003 and December 2019, 229,780 pregnant women gave birth in the Department of Obstetrics in Women’s Hospital. There were 99 cases of placental chorioangioma diagnosed by prenatal ultrasound, an incidence of 0.43‰ (99/229,780). A total of 106 infants were delivered, of which 76 (71.7%) were female and 30 (28.3%) were male. On the basis of the inclusion and exclusion criteria, a total of 54 women were included, of whom 23 who intended to have a vaginal delivery were categorized as the vaginal delivery group, and 31 pregnant women who chose to undergo a cesarean section due to their placental chorioangioma were in the cesarean section group.

The clinical characteristics of the women and their pregnancy outcomes are summarized according to groups in Table 1. There was no significant difference between 2 groups in terms of age, gravidity, parity, BMI, method of fertilization, or the first time of diagnosis of placental chorioangioma. The size of the chorioangioma at delivery in the women between 2 groups was (5.54 ± 1.99 cm vs 6.58 ± 2.09 cm), with no statistically significant difference (*P* = .07). The number of giant choriocarcinoma in the 2 group was no significant differences (87.0% vs 96.8%, *P* = .301). There were no significant differences between groups in the chorioangioma location and blood flow (*P* > .05).

**Table 1:** Baseline characteristics of 2 groups.

	Vaginal delivery group (n = 23)	Cesarean section group (n = 31)	*P* value
Age (yr)*	28.43 ± 3.84	28.42 ± 4.42	0.989
Gravidity†	1 (1–2)	1 (1–2)	0.884
Parity†	1 (1–1)	1 (1–1)	0.542
BMI (kg/m^2^)*	25.80 ± 3.11	26.25 ± 2.79	0.573
Primiparae, n (%)	20 (87.0)	25 (80.6)	0.717
IVF, n (%)	1 (4.3)	0	0.426
Twin pregnancy, n (%)	1 (4.3)	0	0.426
Number of giant chorioangiomas, n (%)	20 (87.0)	30 (96.8)	0.301
CDI characteristics of placental chorioangioma			
Gestational age at diagnosis‡ (weeks)†	34 (28–38)	33 (28–36)	0.194
Chorioangioma location, n (%)			0.090
Placental parenchyma	13 (56.5)	9 (29.0)	
Fetal surface	7 (30.4)	12 (38.7)	
Maternal surface	0	5 (16.1)	
Close to umbilical	3 (13.0)	5 (16.1)	
Blood flow, n (%)			0.132
Avascular	6 (26.1)	5 (16.1)	
Vascularization	14 (60.9)	14 (45.2)	
Rich vascularization	3 (13.0)	12 (38.7)	
Chorioangioma size at the time of delivery (cm)*	5.54 ± 1.99	6.58 ± 2.09	0.070

Note: Normally distributed continuous variables are presented as mean ± SD, while nonnormally distributed variables are presented as median (range).

BMI = body mass index, CDI = color doppler imaging, IVF = in vitro fertilization.

* Mean ± SD.

† Median (range).

‡ Gestational age at diagnosis means the first time of diagnosis of placental chorioangioma during routine prenatal ultrasound examinations.

As shown in Table 2, there was no significant difference between the 2 groups in the incidence of preterm birth, polyhydramnios, premature rupture of membranes, hypertension disorders of pregnancy, or gestational diabetes mellitus (GDM), neonatal ICU admission, neonatal resuscitation. There was no postpartum hemorrhage, puerperal infection and ICU admission in 2 groups. One woman in the vaginal delivery group had a twin pregnancy, so when the neonatal outcomes were analyzed there were 24 fetuses in the group. The 2 groups were similar in terms of birth weight, Apgar scores at 1 minute, incidence of fetal distress and FGR (*P* > .05). There were also no statistically significant differences in other pregnancy outcomes and complications.

**Table 2:** Maternal and perinatal outcome.

	Vaginal delivery group (n = 23)	Cesarean section group (n = 31)	*P* value
Preterm birth, n (%)	2 (8.7)	8 (25.8)	0.161
Polyhydramnios, n (%)	4 (17.4)	5 (16.1)	1.000
Premature rupture of membranes, n (%)	3 (13.0)	2 (6.5)	0.640
Hypertension disorders of pregnancy, n (%)	1 (4.3)	1 (3.2)	1.000
GDM, n (%)	3 (13.0)	4 (12.9)	1.000
Birth weight (kg)*	3.08 ± 0.73	2.97 ± 0.58	0.508
Apgar scores at 1 min†	10 (10–10)	10 (10–10)	0.403
Apgar scores at 5 min†	10 (10–10)	10 (10–10)	0.395
Genda-female, n (%)	15 (62.5)	23 (74.2)	0.391
Term infant, n (%)	21 (87.5)	23 (74.2)	0.314
Fetal distress, n (%)	5 (20.8)	1 (3.2)	0.075
FGR, n (%)	2 (8.3)	2 (6.5)	1.000
Neonatal ICU admission, n (%)	1 (4.2)	1 (3.2)	1.000
Neonatal resuscitation, n (%)	1 (4.2)	1 (3.2)	1.000

Note: Normally distributed continuous variables are presented as mean ± SD, while nonnormally distributed variables are presented as median (range). There was no postpartum hemorrhage, puerperal infection, and ICU admission in 2 groups.

Abbreviations: FGR = fetal growth restriction, GDM = gestational diabetes mellitus.

* Mean ± SD.

† Median (range).

### 3.2. Pregnancy and neonatal outcomes among the women in the vaginal delivery group

As shown in Tables 1 and 3, 82.6% (19/23) of the women in the vaginal delivery group achieved a vaginal delivery. Of the 19 women who successfully delivered vaginally, 17 women had giant chorioangiomas. Regarding the characteristics of the chorioangiomas, 13% were close to the umbilical cord, 56.5% were located on the placenta parenchyma, and 30.4% were located on the fetal surface of the placenta. Placental chorioangiomas were avascular in 26.1% of pregnant women, and 60.9% were vascularized and 13% were richly vascularized. The mean birth weight of the newborns was 3.08 kg, and there were no cases of macrosomia. There were 4 pregnant women who failed to deliver vaginally, 2 had chorioangioma located in the placenta parenchyma with avascularity, one of them was an older primipara (aged > 35 years); 1 had a chorioangioma located close to umbilical cord with avascularity; and 1 had a chorioangioma located on the fetal surface of the placenta with vascularization. The mean diameter of the chorioangiomas of the 4 women who failed to deliver vaginally was 4.25 ± 1.5 cm compared to a mean diameter of 5.82 ± 2 cm of those who deliver had a successful vaginal delivery. Among them, 1 woman converted to cesarean section because of cephalopelvic disproportion, and another woman was delivered by cesarean section because the fetus was in persistent occiput posterior position. The other 2 women underwent cesarean section due to fetal distress during labor. One of them was 35 years old, and had a rupture of membrane and hyperthermia during labor, and the fetal heart rate dropped to 86 beats per minute, through analysis of blood, intrauterine infection was considered, which led to fetal distress, so the cesarean section was performed. The amniotic fluid index of another pregnant woman was 4 cm before delivery, and the fetal heart rate dropped to 86 beats per minute during labor, turned to cesarean section, and the amount of amniotic fluid observed during the operation was only 100 ml.

**Table 3:** The situation of 23 cases of vaginal delivery and 4 cases of vaginal delivery failed to turn to cesarean section.

		Chorioangioma characteristic					Pregnancy outcome					Pregnancy complications
Case	GmPn	Location	Size (cm)	Blood flow (CDI)	Birth process time	Gestational age	Indication of perineotomy or cesarean section	Weigh (g)	Amount of blood during labor (ml)	Gender	Apgar scores*	
1	G3P1	Placenta parenchyma	2.3*2.8*2.5	Vascularization	2h 45m	37 + w	No	3090	150	Male	10	GDM
2	G1P1	Placenta parenchyma	2.9*1.9*2.6	Vascularization	11h 8m	39 + w	No	3750	400	Male	10	No others
3	G2P2	Fetal surface of placenta	4*2.8*1.9	Vascularization	5h 50m	40w	Poor perineal condition	3600	250	Male	10	GDM
4	G3P1	Fetal surface of placenta	3.5*4.0*2.7	Rich vascularization	3h 25m	32 + w	No	1530, 1460	200	Female, Female	10, 9	Twin pregnancy
5	G1P1	Placenta parenchyma	3.9*4.0*4.0	Rich vascularization	5h 20m	38 + w	Fetal distress	2980	250	Male	10	No others
6	G1P1	Placenta parenchyma	4*3*0.5	Vascularization	8h 35m	40 + w	No	3360	150	Female	10	No others
7	G1P1	Fetal surface of placenta	3*4*3	Vascularization	13h 56m	39 + w	Fetal distress	3550	400	Male	10	Fetal distress
8	G2P1	Placenta parenchyma	5*5	Vascularization	8h 57m	30 + w	Fetal distress	1810	200	Male	5	Polyhydramnios, premature rupture of membrane, preterm labor, fetal distress
9	G2P1	Placenta parenchyma	6*4.6*3.2	Vascularization	8h 56m	40 + w	No	3420	200	Male	10	No others
10	G4P2	Close to umbilical	6*5	Vascularization	9h 3m	39 + w	No	3260	150	Female	10	Fetal distress
11	G1P1	Placenta parenchyma	6.1*5.5*3.3	Vascularization	6h 20m	40 + w	No	2460	100	Male	10	FGR
12	G1P1	Placenta parenchyma	6.5*6.9*4.7	Avascular	3h 29m	38 + w	No	2640	200	Female	10	Polyhydramnios
13	G2P1	Fetal surface of placenta	6.8*3.7*5.4	Avascular	6h5m	39 + w	Poor perineal condition	3660	200	Female	10	GDM
14	G4P2	Fetal surface of placenta	7*5.5	Vascularization	7h 10m	39 + w	Fetal distress	3600	440	Female	10	Polyhydramnios
15	G1P1	Placenta parenchyma	7.1*5.6*4.5	Avascular	13h 20m	39 + w	Fetal distress	3250	400	Female	10	Fetal distress
16	G1P1	Fetal surface of placenta	6*8*2.8	Vascularization	10h 15m	38 + w	Poor perineal condition	3750	300	Female	10	No others
17	G1P1	Placenta parenchyma	8*6*4.5	Rich vascularization	26h 10m	40 + w	Poor perineal condition	3350	400	Female	10	Premature rupture of membrane
18	G2P1	Placenta parenchyma	7*8*3	Vascularization	9h 42m	38 + w	No	2480	250	Female	10	FGR
19	G1P1	Close to umbilical cord	10*10	Vascularization	12h 28m	39 + w	No	3950	200	Female	10	Mild preeclampsia, Polyhydramnios, premature rupture of membrane
20†	G3P1	Placenta parenchyma	5.1*5.3*4.3	Avascular	/	39 + w	Fetal distress (FHR slow to 86 bpm)	3050	300	Male	10	Fetal distress, oligohydramnios
21†	G1P1	Fetal surface of placenta	5*4*5	Vascularization	/	40w	Persistent occiput posterior	3600	200	Female	10	NO others
22†	G1P1	Close to umbilical cord	2*2	Avascular	/	40 + w	Cephalopelvic disproportion (protracted active phase)	3600	200	Female	10	NO others
23†	G1P1	Placenta Parenchyma	4.7*4.5*4.3	Avascular	/	38 + w	Fetal distress	3170	150	Female	10	Elderly primipara

* Apgar scores mean Apgar scores at 1 minute.

† Four cases of vaginal delivery failed to turn to cesarean section.

Abbreviations: FGR = fetal growth restriction, GDM = gestational diabetes mellitus.

## 4. Discussion

We compared the clinical characteristics and pregnancy outcomes of women with placental chorioangiomas without obstetric indications for cesarean section by different modes of delivery, and found that it might be safe for pregnant women with placental chorioangiomas to attempt vaginal delivery in the absence of other obstetric indications for cesarean section.

Placental chorioangioma is the most frequent nontrophoblastic benign tumor of the placenta, reported to be present in about 1% of pregnancies.^[13]^ Another study found that the incidence of placental chorioangioma was 0.61% and the fetus was female in 72.2% of the pregnancies.^[14]^ Similarly, Wou et al^[15]^ reported that the fetus was female in 16/23 (69.6%) cases of chorioangioma. The incidence of placental chorioangioma in our study was 0.43‰, fetuses of which 71.7% were female, and the proportion was consistent with that reported in the literature.

Most placental chorioangiomas are asymptomatic; however, giant chorioangiomas (>4 cm) are often associated with adverse pregnancy outcomes such as polyhydramnios, preterm labor, placental abruption, preeclampsia, FGR, fetal anemia, congestive heart failure, fetal nonimmune hydrops, and even fetal death.^[12,16,17]^ There were 4 cases (17.4%) of polyhydramnios in the vaginal delivery group in our study, compared to 5 (16.1%) in the cesarean section group. These results were consistent with a study by Ropacka-Lesiak et al that found an incidence of 14 to 28% in clinical cases.^[18,19]^ FGR has been reported as a consequence of placental chorioangioma by several researchers.^[16]^ Two cases of FGR per group in our study (7.4%) were similar to those previously reported (7.7%).^[19]^ Other researchers have suggested that the vascularity of the tumor may be an independent risk factor for fetal complications regardless of the tumor size.^[20]^ Several scholars also reported that maternal or fetal complications are predominantly associated with large chorioangiomas developing close to the umbilical cord insertion and are associated with fetal death in up to 30% of cases.^[21]^ Only 14.8% of placental chorioangiomas in our study were located near the umbilical cord. This is lower than in previous reports,^[9]^ and may account for the good pregnancy outcomes of the women in our study. The mean chorioangioma size of women in the vaginal delivery group did not differ significantly from that of those in the cesarean section group, demonstrating that women with chorioangiomas might attempt a vaginal delivery, even with giant chorioangiomas.

The simple placental chorioangioma is not the indication for cesarean section, but its severe maternal or fetal complications during pregnancy could be indeed an indication. Some researchers have reported that the fetal condition may deteriorate and leading to delivery by cesarean section, because of fetal growth restriction and the reduced short-term variability on the cardiotocograph.^[13]^ Wu et al^[19]^ also reported a low incidence of spontaneous vaginal deliveries in women whose pregnancies were complicated by chorioangiomas: 27 of 28 patients (96.4%) had cesarean sections while only 1 patient delivered spontaneously, and they usually recommended cesarean section unless pregnant woman were in labor spontaneously for giant placenta chorioangioma. In contrast, Wou et al^[15]^ showed that of 23 pregnant women with placental chorioangioma, only 5 terminated by cesarean section. Similar results were found in the article by Bashiri et al,^[22]^ 9 of 12 women (75.0%) with placental chorioangioma were delivered vaginally.

Up to date, the safety of vaginal delivery is uncertain among women without any obstetric indications for cesarean section, meanwhile this has not been studied in the literature about the association between the placental chorioangiomas and mode of delivery. Therefore, we aimed to determine whether there was a significant difference in the pregnancy outcomes according to the delivery mode among women with placental chorioangioma without obstetric indications for cesarean section. Our results might contribute evidence on the safety of vaginal delivery for these women. In the study, there was no difference between the women who underwent a vaginal delivery and those who chose cesarean section in terms of the maternal characteristics, the CDI characteristics of the placental chorioangiomas, and the pregnancy outcomes. This suggest that many women who chose cesarean section might undergo a trial of vaginal delivery. In addition, our study surprisingly found that some women with giant chorioangiomas could also undergo vaginal delivery if there are no serious complications, although giant placental chorioangioma can cause a range of complications. In our study, 82.6% of pregnant women elected to deliver vaginally were successful and there were only 4 cases of vaginal delivery failure necessitating cesarean section. Some researchers have reported that pregnant outcomes were associated with chorioangioma location and blood flow.^[21,23]^ Although 1 of our 4 cases had a placental chorioangioma located near the umbilical cord and 1 case of vascularization, the direct cause of the vaginal delivery failure among the 4 women with failed vaginal deliveries was fetal distress, persistent occiput posterior fetal position and cephalopelvic disproportion. One case of fetal distress was caused by intrauterine infection and the other was caused by oligohydramnios. Placental chorioangiomas may not be the direct cause of fetal distress. One of the women with a failed vaginal delivery was an older woman (>35 years) in her first pregnancy; however, the risk of complications of vaginal delivery among older women during their first pregnancy is relatively high. Even women with placental chorioangiomas who do not have other obstetric complications, more intensive monitoring is required during vaginal delivery than other women.

This study differs from previous studies in that we had a single indication for cesarean section and no other influencing factors by selecting pregnant women without other obstetric complications for the study. Hence, we believe that the results of this study may provide a base for future studies related to the safety of vaginal delivery for pregnant women with placental chorioangiomas without obstetric indications for cesarean section.

Our study had some limitations. This was a retrospective single-center pilot investigation and women were recruited from only 1 hospital. In addition, we summarized all pathologically confirmed cases of placental chorioangiomas, but the sample size was small. In the future, randomized controlled trials are needed to confirm our results.

## 5. Conclusions

Our data indicated that among women with placental chorioangioma, the pregnancy outcomes did not differ according to the mode of delivery in those without other indications for cesarean section. These pregnant women might attempt vaginal delivery, even if the giant placental chorioangioma. The pregnancy outcomes in different mode of delivery might be similar. If there are risk factors of vaginal delivery failure, the progress of labor needs to be closely monitored. Our results may encourage others to allow women with a low risk of complications to deliver vaginally, and reduce the incidence of cesarean section in women with placental chorioangioma. However, detailed discussions with the patients should take place prior to vaginal delivery regarding the benefits, risks and alternatives.

## Author contributions

LBH contributed to the study conception, the study design, and the drafting of the manuscript. ZJS contributed to the data collection and analysis, and the writing of the manuscript. DWM, CQ, and BXX contributed to the data acquisiton and the critical revision of the manuscript. All authors approved the final manuscript.

## Acknowledgment

We thank the pregnant women for their participation in our study.
